# Disentangling accelerated cognitive decline from the normal aging process and unraveling its genetic components: A neuroimaging-based deep learning approach

**DOI:** 10.21203/rs.3.rs-3328861/v1

**Published:** 2023-09-08

**Authors:** Yulin Dai, Hsu Yu-Chun, Brisa S. Fernandes, Kai Zhang, Li Xiaoyang, Nitesh Enduru, Andi Liu, Astrid M Manuel, Xiaoqian Jiang, Zhongming Zhao

**Affiliations:** The University of Texas Health Science Center at Houston; The University of Texas Health Science Center at Houston; The University of Texas Health Science Center at Houston; The University of Texas Health Science Center at Houston; The University of Texas Health Science Center at Houston; The University of Texas Health Science Center at Houston; The University of Texas Health Science Center at Houston; The University of Texas Health Science Center at Houston; The University of Texas Health Science Center at Houston; The University of Texas Health Science Center at Houston

**Keywords:** Alzheimer’s disease, cognitive decline, dual-loss Siamese ResNet network, polygenic risk score, neuroimaging, genome-wide association studies

## Abstract

**Background:**

The progressive cognitive decline that is an integral component of AD unfolds in tandem with the natural aging process. Neuroimaging features have demonstrated the capacity to distinguish cognitive decline changes stemming from typical brain aging and Alzheimer’s disease between different chronological points.

**Methods:**

We developed a deep-learning framework based on dual-loss Siamese ResNet network to extract fine-grained information from the longitudinal structural magnetic resonance imaging (MRI) data from the Alzheimer’s Disease Neuroimaging Initiative (ADNI) study. We then conducted genome-wide association studies (GWAS) and post-GWAS analyses to reveal the genetic basis of AD-related accelerated cognitive decline.

**Results:**

We used our model to process data from 1,313 individuals, training it on 414 cognitively normal people and predicting cognitive assessment for all participants. In our analysis of accelerated cognitive decline GWAS, we identified two genome-wide significant loci: *APOE* locus (chromosome 19 p13.32) and rs144614292 (chromosome 11 p15.1). Variant rs144614292 (G>T) has not been reported in previous AD GWA studies. It is within the intronic region of *NELL1*, which is expressed in neuron and plays a role in controlling cell growth and differentiation. In addition, *MUC7* and *PROL1/OPRPN*on chromosome 4 were significant at the gene level. The cell-type-specific enrichment analysis and functional enrichment of GWAS signals highlighted the microglia and immune-response pathways. Furthermore, we found that the cognitive decline slope GWAS was positively correlated with previous AD GWAS.

**Conclusion:**

Our deep learning model was demonstrated effective on extracting relevant neuroimaging features and predicting individual cognitive decline. We reported a novel variant (rs144614292) within the *NELL1* gene. Our approach has the potential to disentangle accelerated cognitive decline from the normal aging process and to determine its related genetic factors, leveraging opportunities for early intervention.

## Background

Alzheimer’s disease (AD) is a progressive and degenerative disease of the brain affecting the daily activities of the aging population. Approximately 6.2 million people in the US currently live with AD and the number of individuals with AD is predicted to double by 2025. Cognitive decline and memory impairment are the prominent symptoms of AD [1]. Late-onset AD (LOAD) heritability is as high as 79% [2–5]. Despite the fact that the genetic architecture of LOAD has been identified using millions of participants [6, 7], currently, there is no effective treatment for preventing the development of AD [8, 9]. One of the reasons for this lack of proper identification and effective treatments is that we do not have a coherent and actionable system capable of accurately detecting AD and untangling its effects from the normal aging process. The widely-used Mini-Mental State Examination (MMSE) and Alzheimer’s Disease Assessment Scale-Cognitive Subscale (ADAS-Cog) are strongly influenced by the individual status and non-cognitive domains, such as language, levels of literacy, and cultural and ethical norms [10]. Furthermore, fluctuations in the MMSE and ADAS-Cog tests might lack components sensitive to identifying early-stage dementia, especially mild cognitive impairment (MCI) [11–13], hindering accurate cognitive assessments and leading to misclassification due to test-specific biases. As AD is a brain-related disease, neuroimaging has become one of the main tools to identify the brain structural alterations of memory decline and tackle the progression to AD [14–18]. Alteration in the hippocampus assessed by magnetic resonance imaging (MRI) can occur simultaneously with the first time of amyloid deposition, as early as 18 years prior to dementia [9]. Yet, neuroimaging studies [19] have focused mostly on the conversion from MCI to AD [20]. Van Loenhoud et al. analyzed the differences between predicted brain damage on neuroimaging and cognitive testing. They found that less brain damage than expected was a predictor of lower conversion from normal to MCI or AD, but they did not provide prediction at the individual level [21]. Liu et al. treated the transition as a regression problem, which did not use longitudinal information [22]. In addition, longitudinal MRI has also been used for the prediction of brain age, highlighting the accelerated biological aging in individuals who develop AD dementia [23]. Although much progress has been made recently, there is still main challenges on effectively unraveling the cognitive decline attributed to normal aging effects from those linked to AD [23–26].

Recently, deep learning-based approaches such as the convolutional neural network (CNN) have become popular for brain imaging data analysis, including image classification, abnormality detection, and even early diagnosis of various diseases [27]. These approaches have two advantages: 1) they can process large amounts of data quickly and accurately, and 2) they can detect patterns or features in the complex data that are invisible to the human eye. Magnetic Resonance Imaging (MRI) is a medical imaging technique that is used to diagnose and monitor a variety of medical conditions. A variety of research studies have shown that CNN can be used to diagnose AD by accurately classifying the different stages of dementia using MRI data [28–31]. Despite the advancements in deep learning applications, there is a noticeable research gap in the development of its methodology in large-scale MRI studies. This deficiency could lead to significant overfitting issues and poor generalizability. The absence of a generalizable research that is robust to label bias underscores the need for our study, which proposes to address this gap by transforming supervised prediction problems such as AD versus cognitively normal (CN) into a self-supervised contrastive learning problem, which might have a better power to solvethe current limitations in the field. .

In this study, we combined neuroimaging, clinical, and genetics data to create a comprehensive deep learning-based method for disentangling accelerated cognitive decline from the normal aging process and explore its underlying genetics basis. Our approach, tested on ADNI cohorts, proved to be superior to traditional methods, by uncovering new loci and genes not identified in previous AD studies. Our work presents three major contributions. First, we created pairs of data involving T1-weighted MRI (T1w MRI) and corresponding ADAS-Cog13 neuropsychological assessment results for all possible combinations of time points within the set. These data pairs were then trained with a dual-loss Siamese ResNet model to assess whether a pair of MRI images and cognitive score alterations exceed a certain normal aging threshold. We applied the pre-trained model to predict aging-related cognitive decline for the population at large. By accounting for the confounding factor of normal aging, this model enhances the statistical power of subsequent genome-wide association studies (GWAS) focused on accelerated cognitive decline. Secondly, we adopted metric learning and multitask learning by combining supervised learning and self-supervised contrastive learning tasks on continuous severity scale of MRIs and cogintive assessment. Our model could use unlabeled data to learn similarities and disimilatires between pairs of MRI images, resulting in a robust vector representation of an MRI image which is not dependant on the ground-truth label. Therefore, the learned image representations are robust to label bias and are more generalizable. Tests conducted on ADNI cohorts encompassing CN, MCI, and AD individuals demonstrated that our model outperforms ADAS-Cog13 items, as evidenced by reduced standard error and dispersion measures in the cognitive decline rate. Lastly, our GWAS and subsequent post-GWAS analyses successfully identified novel loci and genes that had remained undiscovered in previous AD GWAS studies.

## Methods

### Alzheimer’s Disease Neuroimaging Initiative (ADNI) data

In this study, we used the ADNI database (ADNI 1, GO, 2, 3) to build the imaging-cognitive score model. The longitudinal analysis of T1w MRI data was used to provide brain structural information of both gray and white matter to track and evaluate brain structural change along the time axis as the disease progresses. We paired T1w MRI images from 1,313 participants with their cognitive score tests assessed from 2003 to 2019; the age of the participants covered a wide spectrum ranging from 55 years old to 91 years old. The 1,313 participants were categorized as CN, MCI, or AD based on their cognitive status at the baseline screening for training the deep learning model. ADNI demographic information is provided in the supplementary table 1 (**Table S1**).

### ADAS-Cog assessment

For the cognitive score assessment, we used the ADAS-Cog13 items scores from ADNI clinical data. ADAS-Cog13 was developed to be used as an index of global cognition in disease progression assessment. ADAS-Cog13 includes 13 items assessing cognitive function [32]. The tasks are related to memory, language, praxis, orientation, number cancellation, and delayed free recall with a total score of 85 points, with higher scores denoting worse performance.

### Image preprocessing

We curated longitudinal 6,711 T1w MRI images from 1,313 participants with paired ADAS-Cog 13 assessments and processed them using Clinica [33] to the Brain Imaging Data Structure (BIDS) format [34]. MRI images were first processed using a nonparametric nonuniform intensity normalization (N3) algorithm [35] to correct the non-uniform intensity. After correction, skull-stripping was performed by PARIETAL [36], followed by registering MRI images to a common template (Montreal Neurological Institute 152) using Freesufer 6.0.1 [37] and removing voxels outside the brain region. All images were prepared in 128 × 160 × 128 resolution and 1.0 mm^3^ voxel size.

### Deep learning pipeline

#### Experimental design

##### Accelerated cognitive decline was defined as having a steeper slope in the cognitive assessment.

**Figure 1:**
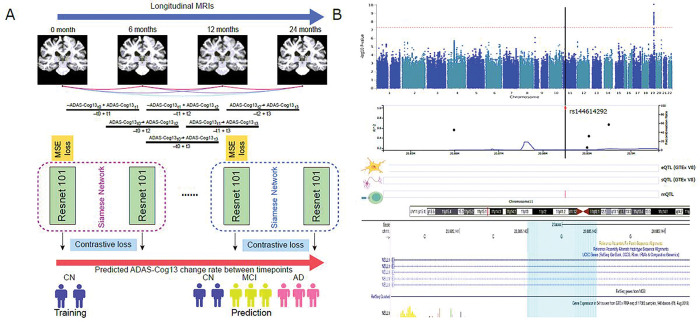
Overview of this work. **A.** Deep learning of accelerated cognitive decline. **B.** GWAS analysis reveals one novel locus related to accelerated cognitive decline.

To calculate the cognitive decline slope, we selected subjects with more than two visits of paired ADAS-Cog13 assessments and T1w MRI scans. Specifically, the ADAS-Cog13 subtest was linked to one T1w MRI scan if it was tested in an interval of 45 days of the T1w MRI assessment. We only included individuals with their diagnosis status either unchanged or having forward transitions among data collection points. To capture a stable cognitive alteration trend, we implemented a time span ranging from a minimum of 6 months to a maximum of 24 months between data collection points to form pairs and excluded subjects that have no more than 2 data points (**Fig. S1**). By applying a skip connection between longitudinal data points, we were able to curate 9,680 data pairs from 1,313 subjects. The median cognitive decline slope ($\frac{ADAS-Cog13t_{i}-ADAS-Cog13t_{i-1}}{t_{i}-t_{i-1}}$, Fig. 1A) across visits was used as the outcome of the following GWAS.

### Deep learning architecture

We illustrated our overall deep learning framework, which employs dual-loss Siamese ResNet, in Fig. 1A. First, the multitask neural network was trained to simultaneously perform two tasks: predicting the actual cognitive score at the first time point of the data pair (regression task) and distinguishing a pair of images belonging to the same/different classes (contrastive learning task). Second, the two tasks share a common backbone neural network structure, which has a similar structure to the Siamese network [38]. The output of the network has two prediction heads with a Multiple Layer Perceptron network structure to perform the two tasks. The model takes paired two separate images from two time points as input, feeds them into the shared subnetworks, and joins the two output embedding vectors to feed into separate task-specific layers [39].

To extract features from MRI data, we used 3D ResNet-101 [40] as subnetworks with shared weights using 3D kernels instead of original 2D kernels. We first introduced mean square error (MSE) loss to counteract baseline differences between pairs, by ensuring the predicted ADAS-Cog13 scores are closely aligned with true target values for the first point of each time pair. We skipped the final fully connected layer and used the high-dimensional vector output to calculate the Euclidean distance between subnetworks. While using the paired image input $X_{1}$ and $X_{2}$, we calculate the Euclidean distance between the subnetwork output vectors $Gw\left(X_{1}\right)$ and $Gw\left(X_{2}\right)$ as $Dw\left(X_{1},X_{2}\right)={∥Gw\left(X_{1}\right)-Gw\left(X_{2}\right)∥}_{2}$. Then, we introduced contrastive loss as $L=(1-Y)Dw^{2}+(Y)\{max(0,m-Dw){\}}^{2}$, where Y is the actual label of a pair of MRI images (Y=0 if belonging to the same class, i.e., no significant change on cognitive score; Y=1 if belonging to different classes, i.e., significant change on cognitive scores). The variable m is a hyperparameter denoting the minimum Euclidean distance (ED) a pair of different-class images should have. In the training analysis, 1,959 data pairs from 289 CN subjects were used, using Adam optimizer [41] and a mini-batch size of 4 to train the model for 200 epochs with an initial learning rate of 10^−4^ and a step-based learning rate scheduler with decay rate γ=0.1 for every 10 epochs on a Nvidia-A100 GPU. In validation, 946 data pairs from 125 CN subjects were used to test the performance of the best model, with minimum validation loss summation of MSE and contrastive loss. Lastly, we predicted all the cognitive decline slopes as changes of ADAS-Cog13 scores divided by time for each subject at each pair as the learned cognitive effect from neuroimaging. Finally, the median of the predicted cognitive decline slope between visits for each subject was used as the covariate of the GWAS.

### Performance evaluation

The normalized predicted error, defined as the difference between predicted and actual ADAS-Cog13 scores divided by time), was used to measure the model performance among dual-loss Siamese ResNet networks with different depths (101, 152, 200) of 3D ResNet subnetwork structures. To verify the stability of our framework, we further compared their GWAS analysis results, including the lead SNPs and Manhattan plots, respectively. We selected our best dual-loss Siamese ResNet model using 3D ResNet-101 as subnetworks and compared the normalized predicted error of model performance with other two existing deep learning methods (Model 1: Ranking convolutional neural network [42], Model 2: Recursive neural network [43].

### Quality control and GWAS

#### Imputation process

We obtained the ADNI raw genotype data from the ADNI [44], including three batches of study (ADNI1 757, ADNI2GO 793, ADNI3 327). We followed the procedure of previous work [45]. Briefly, we first converted all the SNPs to human reference (GRCh37) using liftover [46]. Before Imputation, we performed the standard variants checking procedure to correct abnormal SNPs using the tools developed by the McCarthy group [47]. Then, we submitted all the pre-checked genotype data to the Michigan Imputation Server [48], using the 1000g-phase-3-v5 European ancestry reference panel, respectively. Next, we combined these three cohorts and filtered out those imputed variants with imputation quality < 0.1, the remaining 10,629,535 variants in total.

### Quality control (QC) analysis

We applied KING v.2.2 [49] to remove individuals estimated to be closer than second-degree relatives with a kinship coefficient > 0.0884, which kept 1858 out of 1877 total individuals. ANNOtate VARiation (ANNOVAR) [50] was used to annotate the rsid of each SNP from dbSNP151. Next, we used bcftools [51] and vcftools [52] to replace the ID column of the vcf file. Next, we adopted plink1.9 [53] to conduct the standard QC procedures including, SNP missing rate > 0.02, minor allele frequency > 0.01, and Hardy-Weinberg Equilibrium > 10^−6^. Overall, we obtained 8,836,851 variants for GWAS analysis for 1847 individuals.

### European ancestry (EA) cohort population

The ADNI cohorts are composed of a large proportion of the European ancestry (EA) population. Therefore, we extracted EA subjects by projecting them into the 1000 Genomes Project individuals with different ethnic backgrounds. First, we pruned the SNPs using the command ‘--indep-pairwise 50 5 0.2’ from plink, which greedily pruned 5 pairs of variants in the 50 kb window with a squared correlation greater than 0.2 until no such pairs remained from the window. We downloaded the genotype information of 629 individuals from the 1000 Genomes Project ftp [54]. We selected the previous SNPs after pruning and merging these 629 individuals with our 1858 ADNI participants. We conducted a multidimensional scaling (MDS) analysis to identify the population stratification. We excluded the outliers from EA (**Fig. S2A**). After overlapping with samples with longitudinal MRI data (1290), 1064 individuals with EA were retained for downstream GWAS analysis.

### GWAS for cognitive decline slope

In this work, we explored the genetic variants that contributed to the accelerated cognitive decline slope. We applied two linear regression models to conduct the GWAS analysis on ADNI cognitive decline slope and accelerated cognitive decline slope.

#### Model 1

median Cog decline slope ~ genotype + median predicted aging-related Cog decline slope + PCs + sex + median measured age

#### Model 2

median Cog decline slope ~ genotype + PCs + sex + median measured age, where PCs are the top 10 principal components (PCs) from the multidimensional scaling (MDS) analysis of 1064 genotype data with previous pruned SNPs. Sex information is adopted from the ADNI demographic annotation. The median predicted aging-related Cog decline slope is derived from the pre-trained model as mentioned in the deep learning architecture session. To increase the power and deflated type I error in non-normally distributed quantitative traits, we applied the inverse normal transformation to normalize the median measured age, median cognitive decline slopes, and median predicted cognitive decline slope using r package RNOmni [55].

### Lead SNPs, QTL traits, and colocalization analysis

We defined the lead SNPs with nominal significance (p < 10^−5^). We pruned their nearby SNPs with LD *r*^2^ ≥ 0.6. Then, the remaining SNPs with LD *r*^2^ ≥ 0.1 were pruned to define the independent lead SNPs. These independent loci were combined if they were separated by less than 250 kb.

To understand the potential functions of these variants among different tissues and cell types, we scanned the top three SNPs with r^2^ > 0.4 of the lead SNPs of interest (chr4-rs4694308 and chr11-rs144614292) among thousands of quantitative trait loci (QTL) resources curated in QTLbase v2.2 [56]. We selected the potential QTL traits associated with the SNPs of interest that have significant signals within the high LD region of SNPs of interest. To understand the single-cell QTL in the brain, we adopted the latest brain cell eQTL dataset [57] as well. Colocalization analysis was performed using Bayesian Coloc [58], which aims to identify a genetic variant that has shared causality between expression and GWAS trait. The Coloc script was extracted from the original Coloc package [59]. The posterior probability of H4 > 0.5 was defined as nominal significance.

### Phenotype-wide association studies (PheWAS)

To explore the biological insight of the identified statistically significant variants, we assessed the PheWeb version 1.3.15 [60] to query their impacts in ~ 1400 Phenome-wide association studies (PheWAS) conducted in the UK Biobank cohort. Considering the potential correlation between SNPs within the high linkage disequilibrium region, we checked the top three SNPs with r2 > 0.4 of the lead SNPs of interest (chr4-rs4694308 and chr11-rs144614292).

### Gene-level p-value and over-representative analysis

Gene-level p-value was precalculated by MAGMA [61] (incorporated in FUMA platform) with a 50 kb SNP window surrounding each gene. Then, we performed the gene-set analysis implemented in Functional Mapping and Annotation of GWAS [62, 63], which utilizes a linear regression to test if the conditional (such as gene length and gene correlation) mean association with the cognitive function decline phenotype of genes in curated gene sets is greater than that of genes not in the gene set. The cognitive function gene sets were defined by the 52 genes mapped from all the lead SNPs within 50kb in FUMA platform (**Table S2**). In total, 15,487 gene sets [C2 and Gene Ontology (GO) terms] from Molecular Signatures Database (MSigDB) [64] were used to test the functional over-representation.

### Tissue and cell-type specific enrichment analysis

We adopted the MAGMA tissue-specificity test deployed in FUMA, which performs a linear regression, to test if the cognitive function decline phenotype of genes is more expressed in a specific tissue compared to other tissue types for 53 tissues from GTEx V8 [65].

To understand the cell-type-specificity of the target GWAS genes, we adopted our in-house online tool Web-based Cell-type Specific Enrichment Analysis (WebCSEA) [66]. This platform utilizes our previous deTS algorithm [67] to calculate the raw p-value across 1,355 tissue-cell types curated from the large consortium datasets. A permutation-based test was applied to overcome the potential bias due to the different lengths of signature and type I errors. Specifically, we calculated the permutation p-value by ranking the queried raw p-value over more than the p-values of 20,000 gene lists from GWAS and a rare-variants association study of human complex traits and disease pre-curated in WebCSEA. We adopted the 52 genes mapped from all the lead SNPs within 50kb in FUMA platform to WebCSEA. The suggestive significance was set to 0.001. In addition, we check the tissue and cell type implications of all lead SNPs using our in-house method DeepFun [68], which utilizes the convolutional neural network framework to predict the SNP Activity Difference (SAD) on ~ 8,000 chromatin profiles of 225 tissues or cell types from Encyclopedia of DNA Elements (ENCODE) and Roadmap projects.

### Polygenic risk score (PRS) analysis

LDpred2 [69] was used to conduct polygenic risk score (PRS) calculation. We adopted the summary statistics from the meta-analysis of AD GWAS by Wightman et al [6]. This meta-analysis excluded the proxy cases from UK Biobank and 23andMe subjects, which includes 39,918 cases and 358,140 controls. Only HAPMAP3 variants detected in GWAS summary statistics were used to match with samples’ genotype data. Based on matched SNPs, LDpred2(-grid) was used to calculate the candidate PRS for each individual in the ADNI cohort with each hyperparameter combination. We did the same calculation for different p-value thresholds (1, 0.5, 0.3, 0.1, 0.05). The PRS was generated by selecting the hyperparameter combination that achieves the highest area under the curve (AUC) when using the AD diagnosis as the reference group.

### Two-sample Mendelian randomization analysis

Two-sample Mendelian randomization (2SMR) is a statistical method leveraging independent GWAS summary statistics to evaluate causality between an exposure and an outcome using genetic variants as instrumental variables [70]. Here, we conducted 2SMR analysis to assess the causality of the association between cg07126637 and cognition variation using the R package 2SMR [70]. We first obtained all the methylation qualitative trait locus (mQTL) within the region of cg07126637 from one previous genome-wide mQTL study [71]. Considering that mQTLs may be associated with cg07126637 due to linkage disequilibrium (LD) patterns, we performed LD clumping on mQTLs to remove all SNPs present in the 1000 Genome European population with r^2^ > 0.1 and within 10 kb of the top SNPs. We then extracted and harmonized matched SNPs from our GWAS summary statistics. Finally, we performed 2SMR on the harmonized data using built-in methods in the package, including inverse-variance weighted, Egger, among others.

### Genetic correlation analysis

We calculated the liability-based heritability and the magnitude of genetic correlation between AD and other cognitive function-related phenotypes (**Table S3**) using the LD score regression model [72]. Pre-estimated LD scores were obtained from the 1,000 Genomes Project European reference population, and then we calculated the genetic correlation employing HapMap3 SNPs only with LD reference panel SNPs to minimize potential bias due to differences in LD structure.

## Results

### Deep-learning model can capture the longitudinal impact of neuroimaging on cognitive score

**Figure 2:**
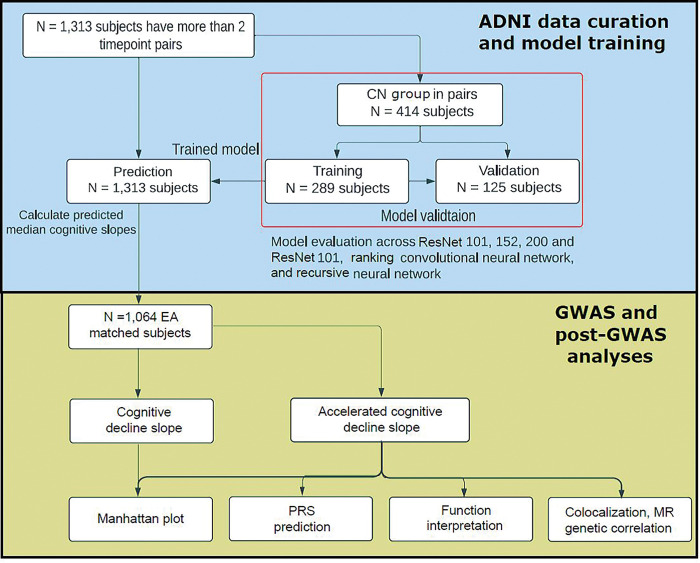
Workflow of the deep learning model design of accelerated cognitive decline prediction using ADNI data and GWAS/post-GWAS analysis.

**Figure 3:**
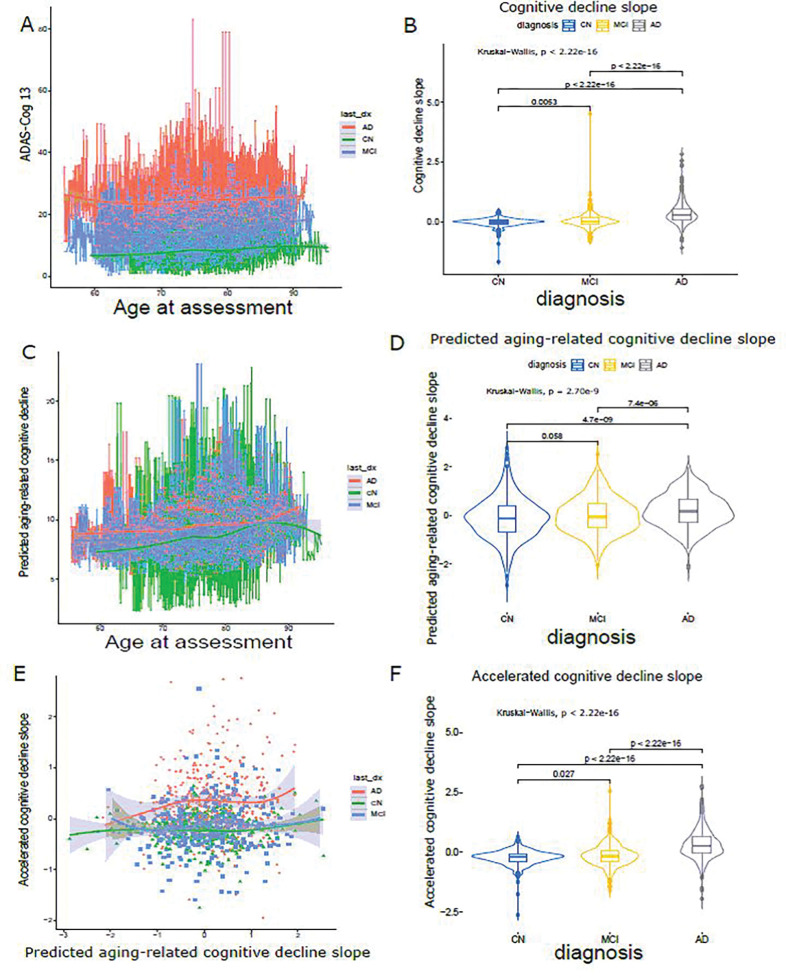
Cognitive decline and cognitive decline slope visualization. **A** longitudinal ADAS-Cog13 assessment for each individual by age at measurement stratified by clinical diagnosis. Each dot represents one measure. **B** Raw cognitive decline slope distribution by clinical diagnosis. **C**. Predicted longitudinal aging-related cognitive decline by age at measurement stratified by clinical diagnosis. Each dot represents one measure. **D** Predicted aging-related cognitive decline slope distribution by clinical diagnosis. **E** Association between predicted aging-related cognitive decline slope and accelerated cognitive decline slope stratified by clinical diagnosis. **F** Accelerated cognitive decline slope distribution by clinical diagnosis. last_dx: last clinical diagnosis.

To disentangle the impact of cognitive decline due to the normal aging process from accelerated aging, we developed a deep learning framework that employs dual-loss Siamese ResNet. This framework enables better prediction of longitudinal cognitive score decline of individuals by extracting the imaging features and leveraging temporal correlations with paired T1w MRIs. We hypothesized that the well-fitted neuroimaging model trained on the population at large can be applied to all subjects to capture the normal cognitive decline due to normal aging. As illustrated in Fig. 1A and Fig. 2, we obtained the matched brain imaging, clinical data (cognitive assessment, ADAS-Cog13), and genotype data. As shown in Fig. 3A, the longitudinal ADAS-Cog13 scores for all 1,313 subjects were considered. We could observe a clear separation among CN, MCI, and AD. We defined the cognitive decline slope between time points $t_{i}$ and $t_{j}$ as $\left(\Delta CS_{t_{i},t_{j}}\right)$ divided by $\left(t_{i}-t_{j}\right)$ (Fig. 3B).

For dual-loss Siamese ResNet, we used 3D ResNet-101 as the subnetworks backbone to extract the paired MRIs $\left(X_{t_{i}},X_{t_{j}}\right)$ data into embedding vector $Gw\left(X_{1}\right)$ and $Gw\left(X_{2}\right)$. Their difference was defined as the Euclidean distance $Dw\left(X_{1},X_{2}\right)={∥Gw\left(X_{1}\right)-Gw\left(X_{2}\right)∥}_{2}$. We leveraged the dual loss design to further capture the similarity/difference between paired MRIs $\left(X_{t_{i}},X_{t_{j}}\right)$. We trained and validated our model on 414 CN individuals in a 70/30 splitting ratio and predicted the cognitive assessment in 1,313 individuals. Model performance was evaluated using the normalized predicted error (NPE, difference of predicted and actual ADAS-Cog13 divided by time) of predicted cognitive decline slopes in the validation cohort (946 pairs from 125 CN individuals). The accelerated cognitive decline slope (Fig. 3C) was calculated as the residual of cognitive decline slope (Fig. 3B) by predicted aging-related cognitive decline slope using linear regression [**Model 1**], see “Methods”). In Fig. 3D, we conducted a pairwise Wilcox test for three clinical diagnoses. Except for CN vs. MCI, which were not significant (p = 0.058), all other comparison groups showed a significant difference.

In addition, the variance of the estimated cognitive decline related to normal aging increased along with the clinical diagnosis, suggesting a larger variation in the CN group, compared to the MCI and AD groups. In the AD group, we uncovered a positive correlation between the aging-related cognitive decline slope and the accelerated cognitive decline slope (Fig. 3E), while no such correlation was observed in the CN or MCI groups. This observation indicates a distinctive effect of the accelerated cognitive decline slope within the AD group. The distribution of the accelerated cognitive decline slopes (Fig. 3F) was also verified in the observations shown in Fig. 3E. In contrast to the original cognitive decline slope across the clinical diagnoses (Fig. 3B), the significance level of accelerated cognitive decline (Fig. 3F) is slightly smaller, indicating that the cognitive decline linked to the accelerated cognitive decline slope exhibits a closer magnitude in comparison to the cognitive decline slope across the diagnosis groups. The significance difference among diagnosis groups is considerably more pronounced in both the cognitive decline slope (Fig. 3B) and the accelerated cognitive decline slope (Fig. 3F) than in the predicted aging-related cognitive decline slope (Fig. 3D). This suggests that cognitive decline associated with normal aging exhibits a smaller magnitude when contrasted with the cognitive decline linked to AD. We further compared our model (NPE = −0.29, σ = 0.0040) with two different deep-learning model designs in **Fig. S3A** (model 1: ranking convolutional neural network [CNN] [42] (NPE = 0.042, σ = 0.0053) and model 2: recursive neural network [RNN] [43] (NPE = −0.34, σ = 0.0040)) and showed that our model has significantly better performance (more constrained error dispersion). Lastly, dual-loss Siamese ResNet with different depths of 3D ResNet Subnetwork [101, 152, 200] show similar performance **Fig. S3B**.

### One novel locus identified by GWAS of accelerated cognitive decline

**Figure 4:**
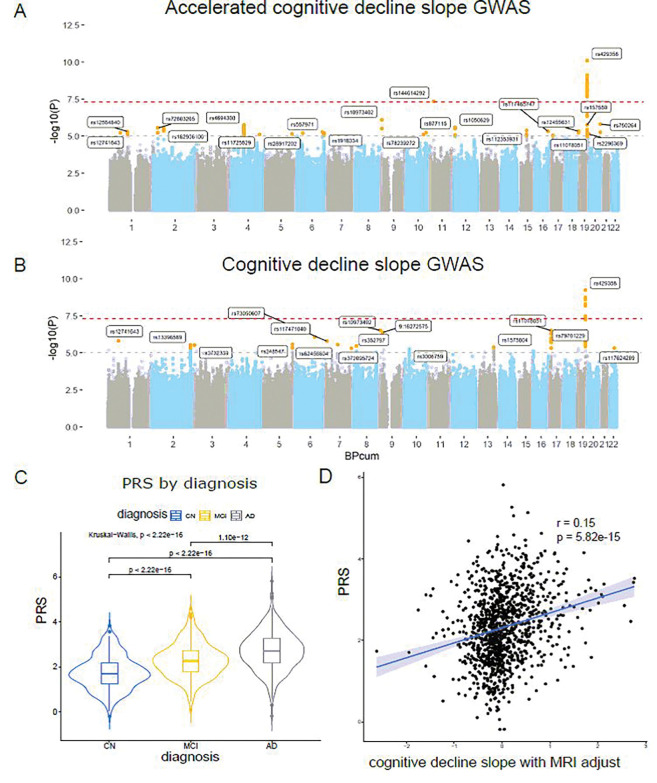
GWAS and PRS analyses. **A.** Manhattan plot for accelerated cognitive decline slope. **B**. Manhattan plot for original cognitive decline slope. The lead SNP in each locus were highlighted. **C**. AD clinical diagnosis by AD PRS distribution. CN: cognitively normal. MCI: mild cognitive impair. **D**. Correlation between AD PRS distribution and accelerated cognitive decline slope.

We formulated two different models to capture the genetic basis that contributes to the cognitive decline slope, an accelerated cognitive decline slope; 2) and the original cognitive decline slope. We followed the illustration in Fig. 1B to conduct a comprehensive post-GWAS analysis to interpret the genetic factors associated with accelerated cognitive decline. As shown in Fig. 4A, the following GWAS for accelerated cognitive decline slope identified two genome-wide significant loci (chr11 rs144614292:G > T p = 3.73 ×10^−8^ and chr19 rs429358 in *APOE* locus). The rs144614292 with a minor allele frequency of 0.05 in EA population is an intronic variant of the *NELL1* gene, which encodes for the teneurin-2 protein and plays a role in synaptogenesis, neurite outgrowth, axon guidance, and neuronal connectivity [73]. In total, we observed 21 nominally significant loci (Table 1). As shown in Fig. 4B, only chr19 *APOE* locus was identified in the original cognitive decline GWAS. We further checked the PRS of AD for these 1,064 individuals using the weight from one previous AD GWAS summary statistics [6]. We identified that the individual PRS is positively correlated with the severity of the clinical diagnosis and is significantly different between diagnostic categories (Fig. 4C). Lastly, we identified that the AD PRS is positively correlated with the normalized cognitive decline slope (Fig. 4D).

### Gene-based value highlights one significant region

We conducted the gene-level p-value using MAGMA [61]. Except for the known *APOE* region genes, we identified two significant genes, MUC7 (1.12 × 10^−6^) and PROL1 (1.42 × 10^−6^), after Bonferroni correction (0.05/19,171 = 2.61 × 10^−6^) in the chr4 lead region locus (rs4694308 C > T) (**Fig. S4A**). This high LD region (r2 > 0.6) expands about 0.14 million bps (chr4:71.26M-chr4:71.40M) and contains five genes (*SMR3A, SMR3B, PROL1/OPRPN, MUC7, AMTN*) directly overlapped with high LD region (**Fig. S5**). Another two genes, (*CABS1* and *AMBN*), are within 100kb of this locus, making it challenging to map the risk variant to the corresponding genes. On the other hand, the original cognitive slope GWAS did not identify other significant genes, except the chr19 *APOE* region (**Fig. S4B**).

### Colocalization and Mendelian randomization

**Figure 5:**
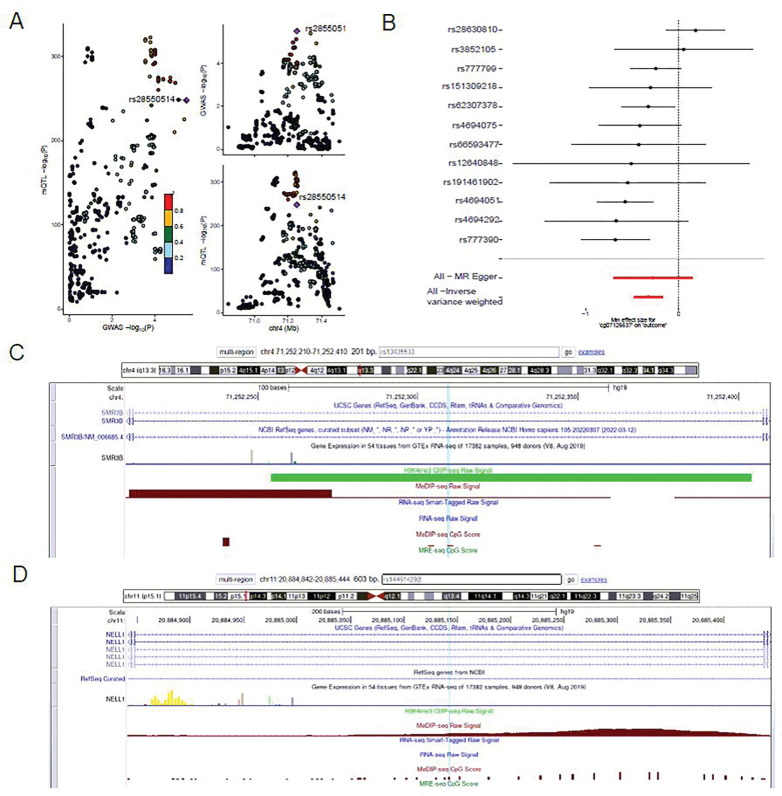
**A.** LocusZoom plot at chr4 rs4694308 C>T locus for exposure mQTL associated with cg07126637 and outcome SNPs associated with accelerated cognitive decline slope. **B.** Mendelian randomization analysis for 12 independent SNPs from 500kb up- and down- stream of rs4694308 between mQTL(cg07126637) and SNPs associated with accelerated cognitive decline slope. All MR Egger results and Inverse variance weighted test were highlighted. **C**. UCSC Genome Browser view of chr4 rs4694308 C>T locus indicated that the merged lead SNP of rs13435533 is impacted by DNA methylation and H3K4me3 signals. **D**. UCSC Genome Browser view of chr11 rs144614292 G>T locus indicated that the lead SNP rs144614292 is impacted by DNA methylation signal.

To verify the novel SNPs and genes findings, we conducted colocalization for two major regions of interest: the gene-level significant locus chr4 rs4694308 C > T (Fig. 5C) and the novel genome-wide significant locus chr11 rs144614292 G > T (Fig. 5D), respectively. We collected single-cell brain-related eQTL dataset [57] and QTL dataset that is hinted by previous QTLbase analysis. We adapted the colocalization method Coloc and QTL data resource (Fig. 5A & Table 2). The only significant PP H4 cg07126637 (0.68) is visualized in the 2SMR analysis (Fig. 5B). A total of 12 SNPs was included in the analysis after LD clumping and harmonization procedures. The results showed that cg07126637 CpG site was significantly associated with cognitive decline slope using the inverse variance weighted method (beta = −0.327, p-value = 5.49 × 10^−5^). There was no horizontal pleiotropy according to the MR Egger regression test (p-value = 0.81). Among the single SNP analysis, rs777390 (GWAS p = 2.89 × 10^−4^) had the most significant results with p-value = 2.76 × 10^−4^.

### Functional interpretation of genetic factors associated with cognitive decline

**Figure 6:**
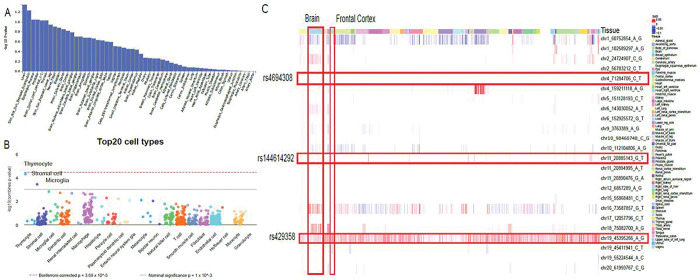
Tissue and cell-type specificity of cognitive decline factors. **A**. Tissue-specificity. **B**. cell-type specificity. The top three general cell types were highlighted. **C** DeepFun shows the SNP Activity Difference (SAD) scores across tissue and cell-types. The lead SNPs of interest and brain tissues were highlighted.

To assess how these genetic factors manifest their effect on tissue and cell types, we applied FUMA MAGMA tissue-specificity test across 53 tissues from GTEx V8 and identified liver, skin, esophagus mucosa, prostate, and brain spinal cord cervical as the top five tissues, although none of them were significant (Fig. 6A). The WebCSEA analysis suggests that thymocyte (combined p-value = 4.93 × 10^−5^), stromal cell (combined p-value = 3.74 × 10^−4^), and microglia (combined p-value = 1.64 × 10^−3^) are the top three cell types related to cognitive decline (Fig. 6B). We applied 21 independent lead SNPs to the DeepFun Web service (Fig. 6C). The chr4 lead SNP (rs4694308 C > T) does not find SNP Activity Difference (SAD), while chr11 lead SNP (rs144614292 G > T) was found to have SAD signals in brain and frontal cortex. Universal SAD alterations could be observed in chr19 lead SNP (rs429358 T > C), suggesting the regulatory effect could impact most tissue and cell types. The functional over-representative analysis of 52 genes mapped from the lead SNPs in MSigDB (C2 and GO terms) highlighted lipid metabolism and immune response functions, aligned with our previous tissue (liver) and cell-type enrichment findings (thymocyte and microglia) (**Fig. S6**). Lastly, no PheWAS conducted within the UK Biobank cohort revealed significant associations (p < 0.05/1419 phenotypes) with the lead SNPs of interest (chr4-rs4694308 and chr11-rs144614292), suggesting no known associations between the two loci with recorded phenotypes (**Fig. S7**).

### Genetic correlation suggests cognitive decline is positively associated with AD

We did a pairwise genetic correlation comparison between the following traits: AD, accelerated cognitive decline slope, original cognitive decline, and educational attainment (**Table S3&S4, Fig. S8**). As expected, AD was negatively correlated with educational attainment (genetic correlation (r_g_) = −012, p = 0.020), which is the only significant r_g_ identified. Positive, although not significant, correlations were observed in AD vs accelerated cognitive decline slope (r_g_ = 0.1, p = 0.65), and Wightman AD vs original cognitive decline (r_g_ = 0.50, p = 0.20). The original cognitive decline vs educational attainment [74] has a light positive correlation (r_g_ = 0.04, = 0.65), while the accelerated cognitive decline vs education attainment has a larger positive correlation (r_g_ = 0.10, p = 0.5), although, again, they were not significant.

## Discussion

We developed a novel deep-learning based approach, leveraging dual-loss Siamese ResNet to learn the normal aging-related cognitive decline slope, and identified the underlying genetic risks for accelerated cognitive decline. Besides the well-known *APOE* region, we identified one genome-wide significant locus (rs144614292, chr11:20885143 G > T) located in the intron region of the gene *NELL1*, which codes the Neural EGFL-like protein 1 (NELL1). The colocalization analysis suggests that this region might be related to mQTL (cg07126637) signal. Moreover, two more genes (*PROL1/OPRPN* and *MUC7*) from chr4 were identified to be gene-level p-value significant. The results of cell-type enrichment and functional analyses indicate that microglia are the most significantly enriched brain cell type, while immune response is the primary biological process associated with these genetic factors.

### Our deep learning model accounts for cognitive decline contributed by normal aging

Our dual-loss Siamese ResNet model is grounded in a series of fundamental assumptions. 1) In stead of learning the supervised prediction problems such as AD versus CN, we assumed our deep learning model could learn the normal aging features from longitudinal CN MRI data by considering the outcome as a continuous metric to enhance its predictive power; 2) We hypothesized that such normal aging features would be distinguishable from AD-related MRI features (the magnitude of alteration in brain regions), therefore allowing us to disentangle AD-related cognitive decline from normal-aging-related cognitive decline; and 3) Within this model, we employed a dual loss framework incorporating both MSE and contrastive loss on pairs of longitudinal MRI and cognitive scores. Conceptually, we postulated that the MSE loss applied to the initial time point of the pair would serve as a baseline, while the contrastive loss would ascertain whether the disparity in MRI images and the change in cognitive scores exceed a predefined threshold for the normal aging effect.

To disentangle normal aging-related cognitive decline features from AD-related cognitive decline, we explicitly trained our model on a CN population and achieved − 0.180 ± 0.261 (mean ± s.d.) on normalized predicted error in the validation set that comprised 946 MRI pairs from 125 subjects. Our model demonstrated superior evaluation performance, with a more constrained dispersion of errors, when compared to the two other designs of deep-learning models (ranking CNN and RNN), indicating that our model can effectively capture the subtle changes of MRI features related to normal aging by using longitudinal neuroimaging data. By employing the insights acquired from the trained model, we were able to differentiate between accelerated cognitive decline associated with AD and age-related cognitive decline, thus providing a more accurate depiction of accelerated cognitive decline with the capacity to better inform the genetic basis of accelerated cognitive decline in subsequent GWAS analyses.

Lastly, we observed a small increase in the slope of accelerated cognitive decline, occurring alongside the rise in the slope of normal aging-related cognitive decline within the CN and MCI groups. On the contrary, a much larger magnitude of increase was observed within the AD group (Fig. 3E). This trend enables the AD group (red) to divergent from the CN (green) and MCI (blue) groups, although all three groups exhibit similar accelerated cognitive decline within the low-end of normal-aging-related cognitive decline. Overall, we quantitatively depict the association between disentangled normal aging-related progress and disease-related progress among diagnosis groups.

### Novel locus and genes

To understand the mechanism underlying this genome-wide significant locus, we explored if the same variant is responsible for the regulatory changes of genes among disease-relevant tissue-cell types. We adapted Bayesian-based Coloc analysis to identify the aligned evidence from publicly available quantitative trait locus (QTL) resources for the human brain and immune cell types. For the lead SNP (rs144614292) in chr11 locus, we identified posterior possibility H4 (0.086) for *NELL1* in excitatory neurons eQTL. For the lead SNP (rs4694308) in chr4 locus, cg07126637 (intron of SMR3B), we identified PP H4 (0.68), suggesting that such “causal” relationship exists in human blood mQTL; another CpG site, cg03970609 (intron of MUC7), does not show a significant colocalization signal. These findings warrant further aging-context brain evidence and experimental validation.

Our GWAS identified three genes related to accelerated cognitive decline, neither of which have been identified as related to AD or cognitive decline in previous GWAS, *NELL1, PROL1,* and *MCU7*; SMR3B was identified by the colocalization analysis. The novel locus in the intron of *NELL1* (rs144614292, p = 3.73 ×10^−8^) has been identified in our GWAS. *NELL1* encodes the protein NEL-like protein 1 (NELL1), a cytoplasmic protein that contains neural epidermal growth factor (EGF)-like repeats. *NELL1* has cytoplasmic expression in the brain, with low brain regional specificity, and is expressed mostly in oligodendrocytes precursor cells and in excitatory and inhibitory neurons. Accordingly, *NELL1* is involved in the modulation of synaptic plasticity via the regulation of its receptor CNTNAP4 (Contactin Associated Protein Family Member 4), which is crucial in synapse development [73]. *NELL1* has been found differentially expressed in the superior temporal gyrus (STG) and inferior frontal gyrus (IFG) of individuals with AD; the STG is a region showing atrophy and epigenetic changes specifically in AD, while the IFG is a region in which atrophy is predominantly related to aging [75]. Interestingly, plasma levels of the protein encoded by *NELL1* are dysregulated in the earliest stage of AD, suggesting the protein coded by *NELL1* is a potential biomarker for early MCI and AD diagnosis [76]. The other two genes identified in our GWAS are *PROL1* and *MUC7*. Gene *PROL1*, also called *OPRPN*, encodes the protein opiorphin prepropeptide, a potent endogenous inhibitor of neprilysin, which crosses the blood-brain barrier [77]. Neprilysin is the central Aβ peptide-degrading enzyme in the brain and it becomes down-regulated and inactive not only during the early stages of AD but also in normal aging. Thus, *PROL1* overexpression might be related to cognitive decline in general by inhibiting neprilysin and thus propitiating amyloid beta accumulation [78]. It has also been hypothesized that opiorphin might act as an antidepressant by activating both μ and δ opioid receptors indirectly [79]. Gene *MUC7* encodes the protein mucin-7 and has been implicated in cholesterol metabolism [80]. Increased serum levels of cholesterol have been identified as a risk factor for AD [81]. Gene *SMR3B* encodes the Submaxillary Gland Androgen Regulated Protein 3B, which overexpresses in the salivary gland, testis, and pituitary from GTEx Portal [65]. Although *SMR3B* was identified has significant PP in Coloc analysis for mQTL blood data, no direct evidence has linked *SMR3B* to cognitive decline or AD yet.

Our work has several limitations. First, we acknowledge that AD-related and normal-aging-related cognitive decline will have shared region of atrophy but different pattern and magnitude [82, 83], which will slightly reduce the accuracy of the predicted normal-aging-related cognitive decline. As shown in Fig. 3E, we did observe a positive association between the accelerated cognitive decline slope and predicted aging-related cognitive decline slope within the AD group. In future, we will explicitly use the non-overlapping regions of atroghy for training our model as proposed by one recent study [84]. From the genetic correlation, we observed a weak positive correlation (r_g_ = 0.10, p = 0.5) between accelerated cognitive decline vs education attainment, albeit not significant, which is not as we expected; it might have been raised from opposite effect directions across shared genetic variants, which might mask overall genetic correlation. Another limitation is that the age of the included participants was smaller for those who were CN than for those who had MCI or AD. Our analyses rest on the assumption that normal aging MRI features are different from AD-related MRI features, which might not necessarily be the case. The rs144614292-chr11 is a multiallelic SNP (G > A / G > T). We adapted its main genotype (G/A) in the GWAS analysis. However, this SNP is not recorded in most QTL databases, including GTEx, due to its multiallelic nature. Therefore, we only identified a weak H4 PP in excitatory neurons in single-cell eQTL study [57]. We expect more solid associations will be identified with more comprehensive eQTL coverage. Lastly, due to the uniqueness of ADNI dataset, no existing dataset has the exact same modalities. In the future, we will incorporate more datasets, such as ANMerge [85], and use Z-score transformed-based method to make the clinical measurement comparable.

## Conclusion

Our new model has successfully extracted detailed information from MRI scans and was superior to cognitive evaluations alone. We provided a quantitative depiction of the relationship between disentangled normal aging progression and disease-related advancement in diagnosis groups. We discovered a significant novel locus (rs144614292) situated in the intronic region of *NELL1*. A colocalization analysis pinpointed *SMR3B*, located in another locus with significant mapped genes *PROL1* and *MUC7*. Our technique exhibits promise in distinguishing accelerated cognitive decline from normal aging, pinpointing its genetic determinants, and providing improved prognostication and management of cognitive decline in patients. This paves the way for potential early intervention strategies.

## Figures and Tables

**Table 1:** List of lead SNPs with p < 1×10^−5^

Locus	rsID	chr	pos	ref	alt	beta	se	p
1	rs12741643	1	60752854	A	G	0.19	0.042	5.90×10^−6^
2	rs12564840	1	102589297	A	G	0.27	0.059	4.91×10^−6^
3	rs72803265	2	24724907	C	G	0.49	0.11	2.79×10^−6^
4	rs182956100	2	56783212	C	T	0.24	0.05	2.84×10^−6^
**5**	**rs4694308**	**4**	**71284706**	**C**	**T**	**0.27**	**0.057**	**1.83×10^−6^**
6	rs11725529	4	159211118	A	G	−0.72	0.16	7.65×10^−6^
7	rs28917202	5	151128193	C	T	−0.68	0.15	7.00×10^−6^
8	rs557971	6	143030052	A	T	−0.61	0.13	5.37×10^−6^
9	rs1918334	6	152925572	G	T	0.22	0.049	6.13×10^−6^
10	rs10973402	9	3763389	A	G	−0.22	0.045	7.93×10^−7^
11	rs78239272	10	98460748	C	G	0.90	0.20	8.02×10^−6^
12	rs877115	10	112104806	A	G	−0.21	0.046	6.02×10^−6^
**13**	**rs144614292**	**11**	**20885143**	**G**	**T**	**−0.67**	**0.12**	**4.38×10^−8^**
14	rs1050629	12	6857289	A	G	0.65	0.14	2.49×10^−6^
15	rs112353931	15	55868481	C	T	0.76	0.16	4.10×10^−6^
16	rs117465747	16	73587857	G	T	0.62	0.14	4.81×10^−6^
17	rs11078051	17	12057796	C	T	−0.22	0.050	8.72×10^−6^
18	rs12455631	18	75082700	A	G	−0.67	0.15	4.68×10^−6^
19	rs157580	19	45395266	A	G	−0.21	0.045	1.77×10^−6^
**19**	**rs429358**	**19**	**45411941**	**C**	**T**	**−0.30**	**0.046**	**8.02×10^−11^**
20	rs2296369	19	55224544	A	C	−0.30	0.067	7.20×10^−6^
21	rs750264	20	61993767	C	G	−0.36	0.075	1.55×10^−6^

chr: chromosome. pos: position (bp). ref: reference allele. alt: alternative allele. se: standard error. Loci of interest were in bold.

**Table 2:** Summary of colocalization analysis results at the chr4 and chr11 loci

Quantitative traits	Locus	Tissue/cell type QTL	QTL ref.	Coloc PP.H4
cg07126637 chr4:71248757 (intron region of *SMR3B*)	rs4694308 (chr4)	Blood mQTL	34493871	0.68*
cg03970609 chr4:71337664 (intron region of *MUC7*)	rs4694308 (chr4)	Blood mQTL	34493871	0.14
*NELL1*	rs144614292 (chr11)	Excitatory Neurons eQTL	35915177	0.086

* indicates nominal significance of posterior possibility (PP H4 > 0.5) in Coloc analysis.

## Abbreviations

AD: Alzheimer’s disease
ADAS-Cog: Alzheimer’s Disease Assessment Scale-Cognitive Subscale
ADNI: Alzheimer’s Disease Neuroimaging Initiative
ANNOVAR: Annotate Variation
AUC: Area under the curve
BIDS: Brain Imaging Data Structure
CN: Cognitively Normal
CNN: Convolutional neural network
EA: European ancestry
ED: Euclidean distance
EGF: Epidermal growth factor
FUMA: Functional mapping and Annotation of GWAS
GO: Gene Ontology
GWAS: Genome-wide association studies
KING: Kinship-based INference for Gwas
LD: Linkage disequilibrium
LOAD: Late-onset AD
MAGMA: Multi-marker Analysis of GenoMic Annotation
MCI: Mild cognitive impairment
MDS: Multi dimensional scaling
MMSE: Mini-Mental State Examination
mQTL: Methylation quantitative trait loci
MRI: Magnetic resonance imaging
MSE: Mean square error
NPE: Normalized predicted error
PC: Principal component
PheWAS: Phenome-wide association studies
PRS: Polygenic risk scores
QTL: Quantitative trait loci
RNN: Recurrent neural network
T1w-MRI: T1-weighted magnetic resonance imaging
WebCSEA: Web-based Cell-type Specific Enrichment Analysis
2SMR –: Two sample Mendelian randomization

## References

[1] Alzheimer’s disease facts and figures. Alzheimers Dement [Internet]. 2021;17:327–406. Available from: 10.1002/alz.12328 33756057

[2] Gatz M, Reynolds CA, Fratiglioni L, Johansson B, Mortimer JA, Berg S, Role of genes and environments for explaining Alzheimer disease. Arch Gen Psychiatry [Internet]. 2006;63:168–74. Available from: 10.1001/archpsyc.63.2.168 16461860

[3] Sims R, Hill M, Williams J. The multiplex model of the genetics of Alzheimer’s disease. Nat Neurosci [Internet]. 2020;23:311–22. Available from: 10.1038/s41593-020-0599-5 32112059

[4] Kunkle BW, Grenier-Boley B, Sims R, Bis JC, Damotte V, Naj AC, Genetic meta-analysis of diagnosed Alzheimer’s disease identifies new risk loci and implicates Aβ, tau, immunity and lipid processing. Nat Genet [Internet]. 2019;51:414–30. Available from: 10.1038/s41588-019-0358-2 30820047 PMC6463297

[5] Pedersen NL, Gatz M, Berg S, Johansson B. How heritable is Alzheimer’s disease late in life? Findings from Swedish twins. Ann Neurol [Internet]. 2004;55:180–5. Available from: 10.1002/ana.10999 14755721

[6] Wightman DP, Jansen IE, Savage JE, Shadrin AA, Bahrami S, Holland D, A genome-wide association study with 1,126,563 individuals identifies new risk loci for Alzheimer’s disease. Nat Genet [Internet]. 2021;53:1276–82. Available from: 10.1038/s41588-021-00921-z 34493870 PMC10243600

[7] Schwartzentruber J, Cooper S, Liu JZ, Barrio-Hernandez I, Bello E, Kumasaka N, Genome-wide meta-analysis, fine-mapping and integrative prioritization implicate new Alzheimer’s disease risk genes. Nat Genet [Internet]. 2021;53:392–402. Available from: 10.1038/s41588-020-00776-w 33589840 PMC7610386

[8] Hadjichrysanthou C, Evans S, Bajaj S, Siakallis LC, McRae-McKee K, de Wolf F, The dynamics of biomarkers across the clinical spectrum of Alzheimer’s disease. Alzheimers Res Ther [Internet]. 2020;12:74. Available from: 10.1186/s13195-020-00636-z 32534594 PMC7293779

[9] Logue MW, Panizzon MS, Elman JA, Gillespie NA, Hatton SN, Gustavson DE, Use of an Alzheimer’s disease polygenic risk score to identify mild cognitive impairment in adults in their 50s. Mol Psychiatry [Internet]. 2019;24:421–30. Available from: 10.1038/s41380-018-0030-8 29487403 PMC6110977

[10] Arevalo-Rodriguez I, Smailagic N, Roqué I Figuls M, Ciapponi A, Sanchez-Perez E, Giannakou A, Mini-Mental State Examination (MMSE) for the detection of Alzheimer’s disease and other dementias in people with mild cognitive impairment (MCI). Cochrane Database Syst Rev [Internet]. 2015;CD010783. Available from: 10.1002/14651858.CD010783.pub2 25740785 PMC6464748

[11] Kueper JK, Speechley M, Montero-Odasso M. The Alzheimer’s Disease Assessment Scale-Cognitive Subscale (ADAS-Cog): Modifications and responsiveness in pre-dementia populations. A narrative review. J Alzheimers Dis [Internet]. 2018;63:423–44. Available from: 10.3233/JAD-170991 29660938 PMC5929311

[12] Devenney E, Hodges JR. The Mini-Mental State Examination: pitfalls and limitations. Pract Neurol [Internet]. 2017;17:79–80. Available from: 10.1136/practneurol-2016-001520 27903765

[13] Arevalo-Rodriguez I, Smailagic N, Roqué-Figuls M, Ciapponi A, Sanchez-Perez E, Giannakou A, Mini-Mental State Examination (MMSE) for the early detection of dementia in people with mild cognitive impairment (MCI). Cochrane Database Syst Rev [Internet]. 2021;7:CD010783. Available from: 10.1002/14651858.CD010783.pub3 34313331 PMC8406467

[14] Ries ML, Carlsson CM, Rowley HA, Sager MA, Gleason CE, Asthana S, Magnetic resonance imaging characterization of brain structure and function in mild cognitive impairment: a review. J Am Geriatr Soc [Internet]. 2008;56:920–34. Available from: 10.1111/j.1532-5415.2008.01684.x 18410325 PMC2668820

[15] Ledig C, Schuh A, Guerrero R, Heckemann RA, Rueckert D. Structural brain imaging in Alzheimer’s disease and mild cognitive impairment: biomarker analysis and shared morphometry database. Sci Rep [Internet]. 2018;8:11258. Available from: 10.1038/s41598-018-29295-9 30050078 PMC6062561

[16] Stephen R, Liu Y, Ngandu T, Antikainen R, Hulkkonen J, Koikkalainen J, Brain volumes and cortical thickness on MRI in the Finnish Geriatric Intervention Study to Prevent Cognitive Impairment and Disability (FINGER). Alzheimers Res Ther [Internet]. 2019;11:53. Available from: 10.1186/s13195-019-0506-z 31164160 PMC6549301

[17] Counts SE, Ikonomovic MD, Mercado N, Vega IE, Mufson EJ. Biomarkers for the early detection and progression of Alzheimer’s disease. Neurotherapeutics [Internet]. 2017;14:35–53. Available from: 10.1007/s13311-016-0481-z 27738903 PMC5233625

[18] Hutton JS, Dudley J, Horowitz-Kraus T, DeWitt T, Holland SK. Associations between screen-based media use and brain white matter integrity in preschool-aged children. JAMA Pediatr [Internet]. 2020;174:e193869. Available from: 10.1001/jamapediatrics.2019.3869 31682712 PMC6830442

[19] van Maurik IS, Vos SJ, Bos I, Bouwman FH, Teunissen CE, Scheltens P, Biomarker-based prognosis for people with mild cognitive impairment (ABIDE): a modelling study. Lancet Neurol [Internet]. 2019;18:1034–44. Available from: 10.1016/S1474-4422(19)30283-2 31526625

[20] Veitch DP, Weiner MW, Aisen PS, Beckett LA, DeCarli C, Green RC, Using the Alzheimer’s Disease Neuroimaging Initiative to improve early detection, diagnosis, and treatment of Alzheimer’s disease. Alzheimers Dement [Internet]. 2022;18:824–57. Available from: 10.1002/alz.12422 34581485 PMC9158456

[21] van Loenhoud AC, van der Flier WM, Wink AM, Dicks E, Groot C, Twisk J, Cognitive reserve and clinical progression in Alzheimer disease: A paradoxical relationship. Neurology [Internet]. 2019;93:e334–46. Available from: 10.1212/WNL.0000000000007821 31266904 PMC6669930

[22] Liu M, Zhang J, Adeli E, Shen D. Joint classification and regression via deep multi-task multi-channel learning for Alzheimer’s disease diagnosis. IEEE Trans Biomed Eng [Internet]. 2019;66:1195–206. Available from: 10.1109/TBME.2018.2869989 30222548 PMC6764421

[23] Gonneaud J, Baria AT, Pichet Binette A, Gordon BA, Chhatwal JP, Cruchaga C, Accelerated functional brain aging in pre-clinical familial Alzheimer’s disease. Nat Commun [Internet]. 2021;12:5346. Available from: 10.1038/s41467-021-25492-9 34504080 PMC8429427

[24] Fjell AM, McEvoy L, Holland D, Dale AM, Walhovd KB, Alzheimer’s Disease Neuroimaging Initiative. What is normal in normal aging? Effects of aging, amyloid and Alzheimer’s disease on the cerebral cortex and the hippocampus. Prog Neurobiol [Internet]. 2014;117:20–40. Available from: 10.1016/j.pneurobio.2014.02.004 24548606 PMC4343307

[25] Ouyang J, Zhao Q, Adeli E, Zaharchuk G, Pohl KM. Disentangling normal aging from severity of disease via weak supervision on longitudinal MRI. IEEE Trans Med Imaging [Internet]. 2022;41:2558–69. Available from: 10.1109/TMI.2022.3166131 35404811 PMC9578549

[26] Lorenzi M, Pennec X, Frisoni GB, Ayache N, Alzheimer’s Disease Neuroimaging Initiative. Disentangling normal aging from Alzheimer’s disease in structural magnetic resonance images. Neurobiol Aging [Internet]. 2015;36 Suppl 1:S42–52. Available from: 10.1016/j.neurobiolaging.2014.07.046 25311276

[27] Sarvamangala DR, Kulkarni RV. Convolutional neural networks in medical image understanding: a survey. Evol Intell [Internet]. 2022;15:1–22. Available from: 10.1007/s12065-020-00540-3 33425040 PMC7778711

[28] Farooq A, Anwar S, Awais M, Rehman S. A deep CNN based multi-class classification of Alzheimer’s disease using MRI. 2017 IEEE International Conference on Imaging Systems and Techniques (IST) [Internet]. IEEE; 2017. p. 1–6. Available from: https://ieeexplore.ieee.org/abstract/document/8261460

[29] AbdulAzeem Y, Bahgat WM, Badawy M. A CNN based framework for classification of Alzheimer’s disease. Neural Comput Appl [Internet]. 2021;33:10415–28. Available from: 10.1007/s00521-021-05799-w

[30] AlSaeed D, Omar SF. Brain MRI analysis for Alzheimer’s disease diagnosis using CNN-based feature extraction and machine learning. Sensors (Basel) [Internet]. 2022 [cited 2023 Aug 13];22:2911. Available from: https://www.mdpi.com/1424-8220/22/8/2911 35458896 10.3390/s22082911 PMC9025443

[31] Dyrba M, Hanzig M, Altenstein S, Bader S, Ballarini T, Brosseron F, Improving 3D convolutional neural network comprehensibility via interactive visualization of relevance maps: evaluation in Alzheimer’s disease. Alzheimers Res Ther [Internet]. 2021;13. Available from: 10.1186/s13195-021-00924-2 PMC8611898 34814936

[32] Mohs RC, Knopman D, Petersen RC, Ferris SH, Ernesto C, Grundman M, Development of cognitive instruments for use in clinical trials of antidementia drugs: additions to the Alzheimer’s Disease Assessment Scale that broaden its scope. The Alzheimer’s Disease Cooperative Study. Alzheimer Dis Assoc Disord [Internet]. 1997;11 Suppl 2:S13–21. Available from: https://www.ncbi.nlm.nih.gov/pubmed/9236948 9236948

[33] El-Rifai O, Melo MD, Hassanaly R, Joulot M, Routier AM, Thibeau-Sutre E, Clinica: an open-source software platform for reproducible clinical neuroscience studies. MRI Together 2021-A global workshop on Open Science and Reproducible MR Research [Internet]. 2021. Available from: https://hal.science/hal-03513920/document

[34] Gorgolewski KJ, Auer T, Calhoun VD, Craddock RC, Das S, Duff EP, The brain imaging data structure, a format for organizing and describing outputs of neuroimaging experiments. Sci Data [Internet]. 2016;3:160044. Available from: 10.1038/sdata.2016.44 27326542 PMC4978148

[35] Sled JG, Zijdenbos AP, Evans AC. A nonparametric method for automatic correction of intensity nonuniformity in MRI data. IEEE Trans Med Imaging [Internet]. 1998;17:87–97. Available from: 10.1109/42.668698 9617910

[36] Valverde S, Coll L, Valencia L, Clèrigues A, Oliver A, Vilanova JC, Assessing the accuracy and reproducibility of PARIETAL: A deep learning brain extraction algorithm. J Magn Reson Imaging [Internet]. 2021; Available from: 10.1002/jmri.27776 34137113

[37] Fischl B. FreeSurfer. Neuroimage [Internet]. 2012;62:774–81. Available from: 10.1016/j.neuroimage.2012.01.021 22248573 PMC3685476

[38] Chicco D. Siamese neural networks: An overview. Methods Mol Biol [Internet]. 2021;2190:73–94. Available from: 10.1007/978-1-0716-0826-5_3 32804361

[39] Bromley J, Guyon I, LeCun Y, Säckinger E, Shah R. Signature verification using a” siamese” time delay neural network. Adv Neural Inf Process Syst [Internet]. 1993;6. Available from: https://proceedings.neurips.cc/paper/1993/hash/288cc0ff022877bd3df94bc9360b9c5d-Abstract.html

[40] He K, Zhang X, Ren S, Sun J. Deep residual learning for image recognition. Proceedings of the IEEE conference on computer vision and pattern recognition [Internet]. 2016. p. 770–8. Available from: http://openaccess.thecvf.com/content_cvpr_2016/html/He_Deep_Residual_Learning_CVPR_2016_paper.html

[41] Kingma DP, Ba J. Adam: A method for stochastic optimization [Internet]. arXiv [cs.LG]. 2014. Available from: http://arxiv.org/abs/1412.6980

[42] Qiao H, Chen L, Zhu F. Ranking convolutional neural network for Alzheimer’s disease mini-mental state examination prediction at multiple time-points. Comput Methods Programs Biomed [Internet]. 2022;213:106503. Available from: 10.1016/j.cmpb.2021.106503 34798407

[43] Lei B, Liang E, Yang M, Yang P, Zhou F, Tan E-L, Predicting clinical scores for Alzheimer’s disease based on joint and deep learning. Expert Syst Appl [Internet]. 2022;187:115966. Available from: https://www.sciencedirect.com/science/article/pii/S0957417421013178?casa_token=IsnIUkSzx3MAAAAA:OtCTyi5Dyd9G1icYOTquqnGcXg7EbEYJCi3iupBVYYyvId52xFWTkZCGfgumfHjtSfmidY6K-QI

[44] ADNI database [Internet]. Alzheimer’s Disease Neuroimaging Initiative Database. [cited 2021 Oct 16]. Available from: http://adni.loni.usc.edu

[45] Li X, Fernandes BS, Liu A, Lu Y, Chen J, Zhao Z, Genetically-regulated pathway-polygenic risk score (GRPa-PRS): A risk stratification method to identify genetically regulated pathways in polygenic diseases [Internet]. medRxiv. 2023 [cited 2023 Jun 29]. p. 2023.06.19.23291621. Available from: 10.1101/2023.06.19.23291621v1

[46] Hinrichs AS, Karolchik D, Baertsch R, Barber GP, Bejerano G, Clawson H, The UCSC Genome Browser Database: update 2006. Nucleic Acids Res [Internet]. 2006;34:D590–8. Available from: 10.1093/nar/gkj144 16381938 PMC1347506

[47] McCarthy Group Tools [Internet]. McCarthy Group Tools. [cited 2022 Feb 8]. Available from: https://www.well.ox.ac.uk/~wrayner/tools/

[48] Michigan Imputation Server [Internet]. Michigan Imputation Server. [cited 2022 Feb 12]. Available from: https://imputationserver.sph.umich.edu/

[49] Manichaikul A, Mychaleckyj JC, Rich SS, Daly K, Sale M, Chen W-M. Robust relationship inference in genome-wide association studies. Bioinformatics [Internet]. 2010;26:2867–73. Available from: 10.1093/bioinformatics/btq559 20926424 PMC3025716

[50] Wang K, Li M, Hakonarson H. ANNOVAR: functional annotation of genetic variants from high-throughput sequencing data. Nucleic Acids Res [Internet]. 2010;38:e164. Available from: 10.1093/nar/gkq603 20601685 PMC2938201

[51] Li H. A statistical framework for SNP calling, mutation discovery, association mapping and population genetical parameter estimation from sequencing data. Bioinformatics [Internet]. 2011;27:2987–93. Available from: 10.1093/bioinformatics/btr509 21903627 PMC3198575

[52] Danecek P, Auton A, Abecasis G, Albers CA, Banks E, DePristo MA, The variant call format and VCFtools. Bioinformatics [Internet]. 2011;27:2156–8. Available from: 10.1093/bioinformatics/btr330 21653522 PMC3137218

[53] Purcell S, Neale B, Todd-Brown K, Thomas L, Ferreira MAR, Bender D, PLINK: a tool set for whole-genome association and population-based linkage analyses. Am J Hum Genet [Internet]. 2007;81:559–75. Available from: 10.1086/519795 17701901 PMC1950838

[54] Genome Project ftp [Internet]. IGSR: The International Genome Sample Resource. [cited 2022 Mar 25]. Available from: ftp://ftp-trace.ncbi.nih.gov/1000genomes/ftp/release/20100804/ALL.2of4intersection.20100804.genotypes.vcf.gz

[55] McCaw ZR, Lane JM, Saxena R, Redline S, Lin X. Operating characteristics of the rank-based inverse normal transformation for quantitative trait analysis in genome-wide association studies. Biometrics [Internet]. 2020;76:1262–72. Available from: 10.1111/biom.13214 31883270 PMC8643141

[56] Zheng Z, Huang D, Wang J, Zhao K, Zhou Y, Guo Z, QTLbase: an integrative resource for quantitative trait loci across multiple human molecular phenotypes. Nucleic Acids Res [Internet]. 2020;48:D983–91. Available from: 10.1093/nar/gkz888 31598699 PMC6943073

[57] Bryois J, Calini D, Macnair W, Foo L, Urich E, Ortmann W, Cell-type-specific cis-eQTLs in eight human brain cell types identify novel risk genes for psychiatric and neurological disorders. Nat Neurosci [Internet]. 2022;25:1104–12. Available from: 10.1038/s41593-022-01128-z 35915177

[58] Giambartolomei C, Vukcevic D, Schadt EE, Franke L, Hingorani AD, Wallace C, Bayesian test for colocalisation between pairs of genetic association studies using summary statistics. PLoS Genet [Internet]. 2014;10:e1004383. Available from: 10.1371/journal.pgen.1004383 24830394 PMC4022491

[59] Wang G, Sarkar A, Carbonetto P, Stephens M. A simple new approach to variable selection in regression, with application to genetic fine mapping. J R Stat Soc Series B Stat Methodol [Internet]. 2020;82:1273–300. Available from: 10.1111/rssb.12388 37220626 PMC10201948

[60] Gagliano Taliun SA, VandeHaar P, Boughton AP, Welch RP, Taliun D, Schmidt EM, Exploring and visualizing large-scale genetic associations by using PheWeb. Nat Genet [Internet]. 2020;52:550–2. Available from: 10.1038/s41588-020-0622-5 32504056 PMC7754083

[61] de Leeuw CA, Mooij JM, Heskes T, Posthuma D. MAGMA: generalized gene-set analysis of GWAS data. PLoS Comput Biol [Internet]. 2015;11:e1004219. Available from: 10.1371/journal.pcbi.1004219 25885710 PMC4401657

[62] Watanabe K, Taskesen E, van Bochoven A, Posthuma D. Functional mapping and annotation of genetic associations with FUMA. Nat Commun [Internet]. 2017;8:1826. Available from: 10.1038/s41467-017-01261-5 29184056 PMC5705698

[63] Watanabe K, Umićević Mirkov M, de Leeuw CA, van den Heuvel MP, Posthuma D. Genetic mapping of cell type specificity for complex traits. Nat Commun [Internet]. 2019;10:3222. Available from: 10.1038/s41467-019-11181-1 31324783 PMC6642112

[64] Liberzon A, Birger C, Thorvaldsdóttir H, Ghandi M, Mesirov JP, Tamayo P. The Molecular Signatures Database (MSigDB) hallmark gene set collection. Cell Syst [Internet]. 2015;1:417–25. Available from: 10.1016/j.cels.2015.12.004 26771021 PMC4707969

[65] Consortium GTEx. The Genotype-Tissue Expression (GTEx) project. Nat Genet [Internet]. 2013;45:580–5. Available from: 10.1038/ng.2653 23715323 PMC4010069

[66] Dai Y, Hu R, Liu A, Cho KS, Manuel AM, Li X, WebCSEA: web-based cell-type-specific enrichment analysis of genes. Nucleic Acids Res [Internet]. 2022;50:W782–90. Available from: 10.1093/nar/gkac392 35610053 PMC10359109

[67] Pei G, Dai Y, Zhao Z, Jia P. deTS: tissue-specific enrichment analysis to decode tissue specificity. Bioinformatics [Internet]. 2019;35:3842–5. Available from: 10.1093/bioinformatics/btz138 30824912 PMC6761978

[68] Pei G, Hu R, Dai Y, Manuel AM, Zhao Z, Jia P. Predicting regulatory variants using a dense epigenomic mapped CNN model elucidated the molecular basis of trait-tissue associations. Nucleic Acids Res [Internet]. 2021;49:53–66. Available from: 10.1093/nar/gkaa1137 33300042 PMC7797043

[69] Privé F, Arbel J, Vilhjálmsson BJ. LDpred2: better, faster, stronger. Bioinformatics [Internet]. 2021;36:5424–31. Available from: 10.1093/bioinformatics/btaa1029 33326037 PMC8016455

[70] Hemani G, Zheng J, Elsworth B, Wade KH, Haberland V, Baird D, The MR-Base platform supports systematic causal inference across the human phenome. Elife [Internet]. 2018;7:e34408. Available from: https://elifesciences.org/articles/34408 29846171 10.7554/eLife.34408 PMC5976434

[71] Min JL, Hemani G, Hannon E, Dekkers KF, Castillo-Fernandez J, Luijk R, Genomic and phenotypic insights from an atlas of genetic effects on DNA methylation. Nat Genet [Internet]. 2021;53:1311–21. Available from: 10.1038/s41588-021-00923-x 34493871 PMC7612069

[72] Bulik-Sullivan BK, Loh P-R, Finucane HK, Ripke S, Yang J, Schizophrenia Working Group of the Psychiatric Genomics Consortium, et al. LD Score regression distinguishes confounding from polygenicity in genome-wide association studies. Nat Genet [Internet]. 2015;47:291–5. Available from: 10.1038/ng.3211 25642630 PMC4495769

[73] Li C, Zheng Z, Ha P, Chen X, Jiang W, Sun S, Neurexin superfamily cell membrane receptor contactin-associated protein like-4 (Cntnap4) is involved in neural EGFL-like 1 (Nell-1)-responsive osteogenesis. J Bone Miner Res [Internet]. 2018;33:1813–25. Available from: 10.1002/jbmr.3524 29905970 PMC6390490

[74] Okbay A, Beauchamp JP, Fontana MA, Lee JJ, Pers TH, Rietveld CA, Genome-wide association study identifies 74 loci associated with educational attainment. Nature [Internet]. 2016;533:539–42. Available from: 10.1038/nature17671 PMC4883595 27225129

[75] Li QS, De Muynck L. Differentially expressed genes in Alzheimer’s disease highlighting the roles of microglia genes including OLR1 and astrocyte gene CDK2AP1. Brain Behav Immun Health [Internet]. 2021;13:100227. Available from: 10.1016/j.bbih.2021.100227 34589742 PMC8474442

[76] Jiang Y, Zhou X, Ip FC, Chan P, Chen Y, Lai NCH, Large-scale plasma proteomic profiling identifies a high-performance biomarker panel for Alzheimer’s disease screening and staging. Alzheimers Dement [Internet]. 2022;18:88–102. Available from: 10.1002/alz.12369 34032364 PMC9292367

[77] Wisner A, Dufour E, Messaoudi M, Nejdi A, Marcel A, Ungeheuer M-N, Human Opiorphin, a natural antinociceptive modulator of opioid-dependent pathways. Proc Natl Acad Sci U S A [Internet]. 2006;103:17979–84. Available from: 10.1073/pnas.0605865103 17101991 PMC1693858

[78] El-Amouri SS, Zhu H, Yu J, Marr R, Verma IM, Kindy MS. Neprilysin: an enzyme candidate to slow the progression of Alzheimer’s disease. Am J Pathol [Internet]. 2008;172:1342–54. Available from: 10.2353/ajpath.2008.070620 18403590 PMC2329843

[79] Yang Q-Z, Lu S-S, Tian X-Z, Yang A-M, Ge W-W, Chen Q. The antidepressant-like effect of human opiorphin via opioid-dependent pathways in mice. Neurosci Lett [Internet]. 2011;489:131–5. Available from: 10.1016/j.neulet.2010.12.002 21145938

[80] Graham SE, Clarke SL, Wu K-HH, Kanoni S, Zajac GJM, Ramdas S, The power of genetic diversity in genome-wide association studies of lipids. Nature [Internet]. 2021;600:675–9. Available from: 10.1038/s41586-021-04064-3 PMC8730582 34887591

[81] Loera-Valencia R, Goikolea J, Parrado-Fernandez C, Merino-Serrais P, Maioli S. Alterations in cholesterol metabolism as a risk factor for developing Alzheimer’s disease: Potential novel targets for treatment. J Steroid Biochem Mol Biol [Internet]. 2019;190:104–14. Available from: 10.1016/j.jsbmb.2019.03.003 30878503

[82] Bakkour A, Morris JC, Wolk DA, Dickerson BC. The effects of aging and Alzheimer’s disease on cerebral cortical anatomy: specificity and differential relationships with cognition. Neuroimage [Internet]. 2013;76:332–44. Available from: 10.1016/j.neuroimage.2013.02.059 23507382 PMC4098706

[83] Habes M, Janowitz D, Erus G, Toledo JB, Resnick SM, Doshi J, Advanced brain aging: relationship with epidemiologic and genetic risk factors, and overlap with Alzheimer disease atrophy patterns. Transl Psychiatry [Internet]. 2016;6:e775. Available from: 10.1038/tp.2016.39 27045845 PMC4872397

[84] Hwang G, Abdulkadir A, Erus G, Habes M, Pomponio R, Shou H, Disentangling Alzheimer’s disease neurodegeneration from typical brain ageing using machine learning. Brain Commun [Internet]. 2022;4:fcac117. Available from: 10.1093/braincomms/fcac117 35611306 PMC9123890

[85] Birkenbihl C, Westwood S, Shi L, Nevado-Holgado A, Westman E, Lovestone S, ANMerge: A comprehensive and accessible Alzheimer’s disease patient-level dataset. J Alzheimers Dis [Internet]. 2021;79:423–31. Available from: 10.3233/JAD-200948 33285634 PMC7902946

